# Yeast Mitochondrial Translation Initiation Factor 3 Interacts with Pet111p to Promote *COX2* mRNA Translation

**DOI:** 10.3390/ijms21103414

**Published:** 2020-05-12

**Authors:** Ivan Chicherin, Sergey Levitskii, Maria V. Baleva, Igor A. Krasheninnikov, Maxim V. Patrushev, Piotr Kamenski

**Affiliations:** 1 Faculty of Biology, M.V.Lomonosov Moscow State University, Moscow 119234, Russia; i.v.chicherin@gmail.com (I.C.); krolick@yandex.ru (S.L.); mary-bw@mail.ru (M.V.B.); iakrasheninnikov@protein.bio.msu.ru (I.A.K.); 2 Institute of Functional Genomics, M.V.Lomonosov Moscow State University, Moscow 119234, Russia; 3 National Research Centre “Kurchatov Institute”, 1 Akademik Kurchatov Square, 123182 Moscow, Russia; maxpatrushev@yandex.ru

**Keywords:** mitochondria, translation, initiation factor, translational activator

## Abstract

Mitochondrial genomes code for several core components of respiratory chain complexes. Thus, mitochondrial translation is of great importance for the organelle as well as for the whole cell. In yeast, mitochondrial translation initiation factor 3, Aim23p, is not essential for the organellar protein synthesis; however, its absence leads to a significant quantitative imbalance of the mitochondrial translation products. This fact points to a possible specific action of Aim23p on the biosynthesis of some mitochondrial protein species. In this work, we examined such peculiar effects of Aim23p in relation to yeast mitochondrial *COX2* mRNA translation. We show that Aim23p is indispensable to this process. According to our data, this is mediated by Aimp23p interaction with the known specific factor of the *COX2* mRNA translation, Pet111p. If there is no Aim23p in the yeast cells, an increased amount of Pet111p ensures proper *COX2* mRNA translation. Our results demonstrate the additional non-canonical function of initiation factor 3 in yeast mitochondrial translation.

## 1. Introduction

Mitochondria originated from the ancient free-living bacterial species, according to the endosymbiotic theory [1]. In the course of evolution, the vast majority of mitochondrial genes have migrated to the nucleus. However, mitochondrial genomes of modern eukaryotes still code for several proteins, most of which are components of the respiratory chain complexes. Correspondingly, mitochondria have kept their systems of genetic material maintenance and expression.

Mitochondrial translation mechanisms closely resemble those of bacteria. On the other hand, many specific features of this process have been discovered, especially as a result of mitochondrial ribosomes structural studies [2,3]. Many of these features, both structural [4] and mechanistic [5], appear at the initiation step. In particular, mitochondrial initiation factor 3 (mtIF3) shares little similarity with its bacterial counterpart [6]. Consequently, researchers could not identify mtIF3 in several well-characterized species, such as *C. elegans* and budding yeast *S. cerevisiae,* for many years. In 2012, in silico and in vivo analyses validated yeast protein Aim23p as mtIF3 [6]. Aim23p is generally organized in the same way as mammalian mtIF3 according to computer modelling: both proteins consist of core part (which is similar to bacterial IF3) flanked by mitochondria-specific N- and C-terminal extensions [7,8]. *E. coli* IF3 can partially fulfil the functions of Aim23p in yeast mitochondria [5], which is valuable evidence of the latter being *bona fide* initiation factor 3.

Several sets of data point to the peculiarity of Aim23p as the factor of mitochondrial translation. In in vitro study, it has been shown that recombinant Aim23p binds both small and large subunits of yeast mitochondrial ribosomes [9], while a canonical IF3 is known to preferably bind the small one; the latter is also true for mammalian mtIF3 [7,10]. Aim23p also possesses a quite unusual effect on the bacterial ribosomes: it fixes their dynamic state which might be an intermediate state of ribosome dissociation [11]. Again, this is not the case for mammalian mtIF3, which exhibits typical, although quite poor, functional activities with regard to the bacterial ribosomes [12]. Finally, the most interesting Aim23p feature is the effect of its gene deletion on yeast mitochondrial translation. IF3 is an obligatory component of any protein synthesis system studied to date. However, yeast mitochondria can perform translation in the absence of Aim23p. It is worth mentioning that such translation is strongly imbalanced. Robust biochemical analysis suggests that biosyntheses of four out of eight proteins encoded in the mitochondrial genome (namely Var1p, Cox3p, Cob1p, Atp8p) do not depend on Aim23p. On the other hand, steady-state levels of Cox1p and Cox2p decrease, while the amounts of Atp6p and Atp9p become elevated in the absence of Aim23p [13].

The presence of translational activators is another interesting feature of the yeast mitochondrial protein synthesis apparatus [14,15]. These regulatory proteins specifically activate the translation of a particular mRNA, most often via interaction with its untranslated regions (UTRs). Many activators are involved in feedback loop mechanisms that coordinate the biosynthesis of mitochondrial proteins with the assembly of respiratory complexes [16]. The detection of the personalized effect of Aim23p on mitochondrial mRNAs translation led to hypothesizing a functional link between this protein and the system of the translational activators. This work is aimed at the experimental validation of this hypothesis.

We decided to investigate whether Aim23p interacts with the 5′-UTRs of the mitochondrial mRNAs or with the corresponding translational activators. Obviously, one should concentrate on either *COX1* or *COX2* mRNAs, because the amounts of both these proteins decrease in *AIM23∆* background [13], which allows for proposing some direct activating effect of Aim23p on these mRNAs. We have chosen *COX2* mRNA for our experiments since (1) its 5′-UTR is shorter than that of *COX1* mRNA (54 vs. 461 nucleotides [17,18]), which makes it easier to work with; (2) a single activator, Pet111p, is described for *COX2* mRNA [19,20], while at least four are known for *COX1* mRNA [14]. Thus, we aimed to test the physical and genetic interaction of Aim23p with the 5′-UTR of the *COX2* mRNA and Pet111p.

## 2. Results

### 2.1. Aim23p Ensures COX2 mRNA Translation via Its UTRs

**Figure 1:**
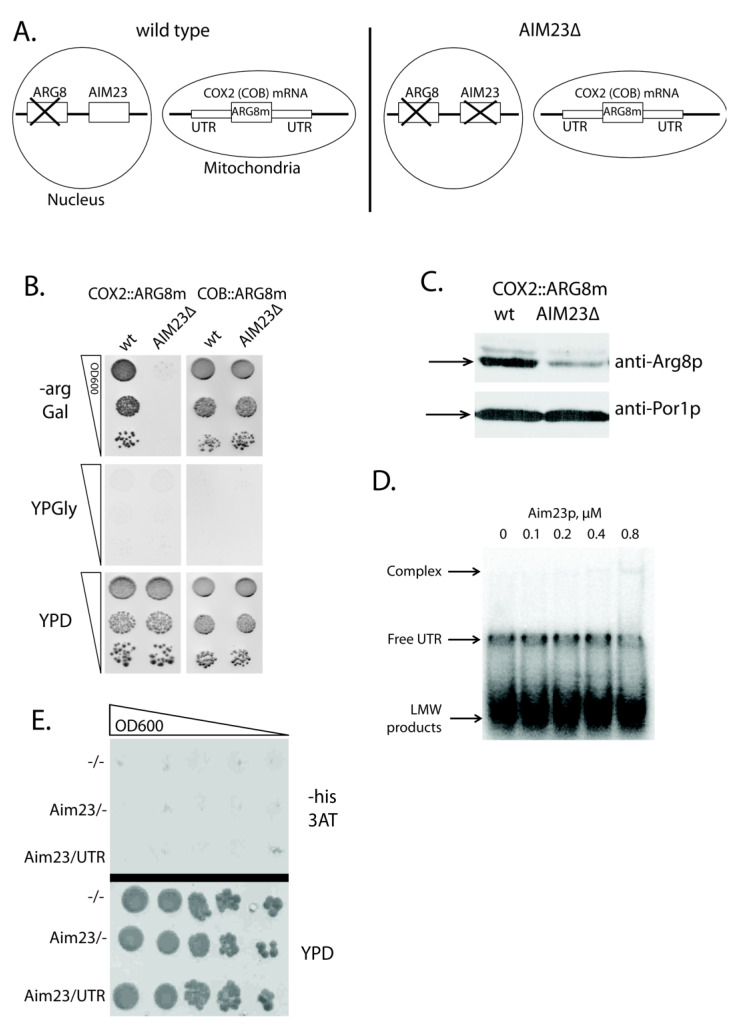
The detection of genetic, but not physical, interaction of Aim23p with untranslated regions (UTRs) of *COX2* mRNA. (**A**) Scheme of nuclear and mitochondrial DNA organization of the yeast strains used in the experiments. Wild type: either HMD22 strain (*COX2::ARG8m* [22]) or MRSI^0^ ΔCOB strain (*COB::ARG8m* [23]). AIM23Δ: the same strains with *AIM23* genomic disruption. (**B**) The plates with different media (indicated on the left: -arg Gal— synthetic arginine-lacking medium with galactose, YPGly—glycerol-containing full medium, YPD—glucose-containing rich medium) after two days of growth of the yeast strains indicated on the top. The ten-fold consecutive dilutions of the yeast suspensions were plated. The experiment was done in three biological replicates; the characteristic picture is presented. (**C**) Western-blot hybridization of mitochondrial proteins isolated from the above-mentioned strains with anti-Arg8p antibodies (top part; kindly provided by Martin Ott). As a loading control, the same samples were blotted with anti-porin 1 antibodies (bottom part; produced by Almabion, Russia). The corresponding protein bands are marked with arrows. The experiment was done in three biological replicates; the characteristic picture is presented. (**D**) Results of the gel-shift experiment. In vitro synthesized *COX2* mRNA 5′-UTR was labeled with ^32^P-ATP and then incubated with different amounts of the recombinant Aim23p (molar concentrations of the protein are indicated on the top). Reaction products were separated using native Polyacrylamide Gel Electrophoresis (PAGE) and visualized by radioautography. The gel zones corresponding to low molecular weight (LMW) products, free UTR and UTR complex with Aim23p are marked by the arrows on the left. The experiment was done in three biological replicates; the characteristic picture is presented. (**E**) Results of the three-hybrid system assay. Three derivatives of the L40 coat yeast strain were analyzed: the first one was transformed by two empty vectors, pACT2 and pIIIA/MS2-1 (−/−); the second one was transformed by pACT2 vector with cloned *AIM23* gene and by empty pIIIA/MS2-1 vector (AIM23/−); the third one was transformed by pACT2 vector with cloned *AIM23* gene and by pIIIA/MS2-1 vector with the cloned sequence of the *COX2* mRNA 5′-UTR (AIM23/UTR). The names of the strains are depicted on the left. Top panel: growth on medium lacking histidine supplemented with the 3-amino-1,2,4-triazole (3-AT). Bottom panel: growth on the rich glucose-containing medium (YPD). The ten-fold consecutive dilutions of the yeast suspensions were plated. The experiment was done in four biological replicates; the characteristic picture is presented.

A good approach exists to access the activator effect of a protein on a given yeast mitochondrial mRNA translation. For that, one should use a mutant yeast strain where: (1) *ARG8* gene (coding for the acetylornithine aminotransferase, the enzyme of arginine biosynthesis pathway) is deleted in nuclear DNA; (2) the open reading frame of mitochondrial mRNA tested is replaced by that of *ARG8* (codon-optimized for mitochondrial expression), while UTRs of this mRNA remain intact [21]. The cells of such strains can grow on the medium lacking arginine. Upon the deletion of the gene studied, failure to grow in the absence of arginine indicates that a protein product of this gene facilitates the translation of target mRNA acting through its UTRs. We have applied this approach to check whether Aim23p takes part in the *COX2* mRNA translation. We had two yeast strains, where *COX2* or *COB* open reading frames were replaced by the mitochondrial version of *ARG8* (further referred to as *ARG8m*) (both strains were kindly provided by Martin Ott). *COB::ARG8m* was used as a control strain, since we showed earlier that the biosynthesis of Cob1p did not depend on Aim23p [13]. Next, we inactivated the *AIM23* gene in these strains by replacing it with KanMX4 cassette (*AIM23Δ* on Figure 1A) and checked the ability of the resulting strains to grow on the arginine-lacking medium (Figure 1B). After two days of incubation, we detected no growth for the strain *COX2::ARG8m AIM23Δ*, while the parental strain containing Aim23p grew normally. This clearly demonstrates that the effect of Aim23p on Cox2p biosynthesis is mediated by UTRs of *COX2* mRNA. On the other hand, both *COB::ARG8m* and *COB::ARG8m AIM23Δ* strains grew equally well on the arginine-lacking medium, which is in perfect agreement with the previously detected independence of Cob1p biosynthesis from Aim23p. All of the tested strains did not grow on the glycerol-containing medium, which was expected: using glycerol as a carbon source is only possible if yeast mitochondria are fully functional which is not the case in our experiment as all strains lack either Cox2p or Cob1p. 

A typical translational activator is absolutely required for the biosynthesis of corresponding mitochondrially-encoded protein, which means that the latter is not synthesized at all in the absence of an activator [15]. Aim23p does not meet this criterion: we detected both Cox1p and Cox2p in *AIM23Δ* strain, although in very small amounts [13]. In present work, we have confirmed this observation by Western-blot analysis of mitochondrial proteins that were isolated from *COX2::ARG8m* and *COX2::ARG8m AIM23Δ* strains with anti-Arg8p antibodies (Figure 1C). In the *COX2::ARG8m AIM23Δ* strain, the amount of Arg8p is significantly less than in the parental strain. However, antibodies still detect the protein, which indicates that Aim23p is not strictly essential for the translation of the *COX2* mRNA. Correspondingly, although Aim23p promotes *COX2* mRNA translation through its UTRs, this protein should not be regarded as a canonical translational activator.

### 2.2. Aim23p Physical Interaction with COX2 mRNA 5′-UTR Is Detectable Neither in Vitro Nor in Vivo

Nevertheless, the possible physical interaction of Aim23p with 5′-UTR of the *COX2* mRNA should be tested. For that, we performed gel-shift experiments with recombinant Aim23p and in vitro synthesized 5′-UTR of *COX2* mRNA in the presence of competitor (total yeast tRNAs) (Figure 1D). We detected no strong interaction: one can see a poorly visible complex band, but only at the maximal Aim23p concentration tested (0.8 µM). We also used the alternative method of this interaction detection, namely yeast 3-hybrid system [24]. This assay allows for detecting the interaction between a protein and an RNA by using specially designed strains and plasmids: if such interaction takes place, an experimental yeast strain would grow on the medium without histidine because of the *HIS3* gene induced expression. We were not able to detect the physical interaction of Aim23p with 5′-UTR of *COX2* mRNA using this method, as shown in Figure 1E. Overall, even if such interaction takes place, it seems to be very weak and, in our opinion, cannot explain the observed effect of Aim23p on the *COX2* mRNA translation.

### 2.3. Aim23p Physically Interacts with Pet111p and Enhances Its Interaction with COX2 mRNA 5′-UTR

**Figure 2:**
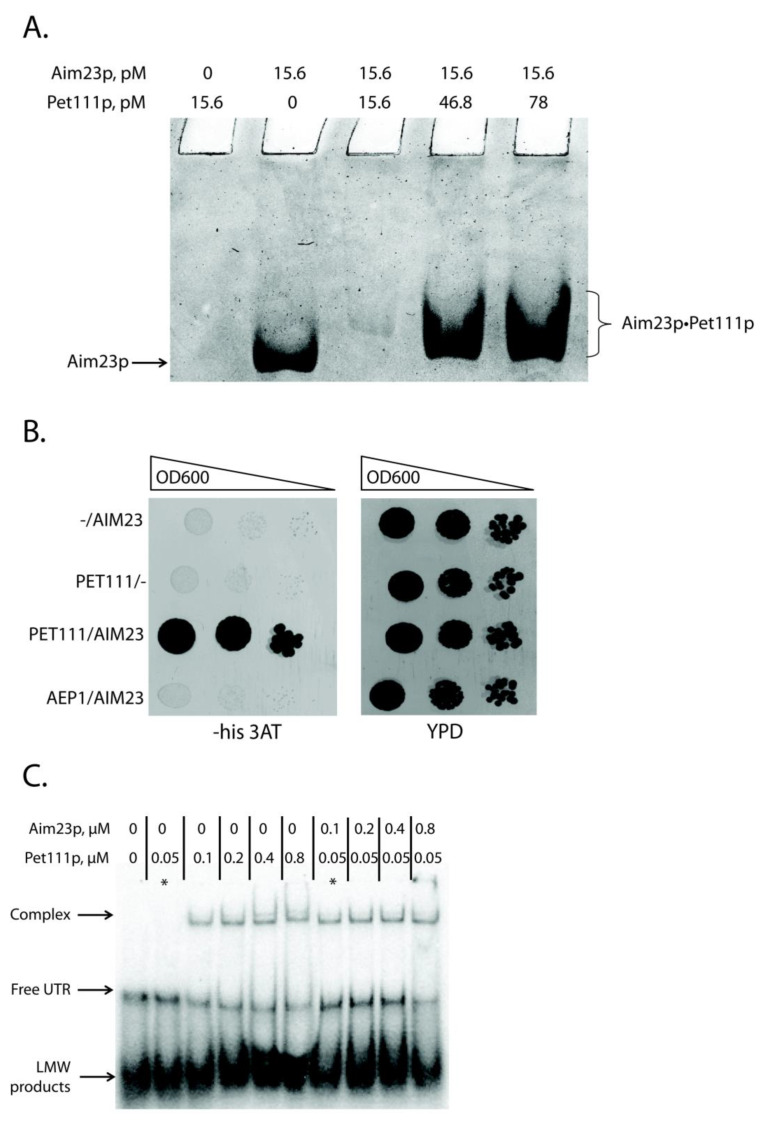
Aim23p physically interacts with Pet111p and helps the latter to bind 5′-UTR of *COX2* mRNA. (**A**) Detection of Aim23p interaction with Pet111p *in vitro*. Recombinant proteins (either one of them or both together) were separated by the native PAGE. The gel zones corresponding to free Aim23p or Aim23p•Pet111p complex are marked on the left and on the right, respectively. The experiment was done in four biological replicates; the characteristic picture is presented. (**B**) Results of the two-hybrid system assay. Four derivatives of the Y190 yeast strain were analyzed, transformed by: (1) empty pAS2ΔΔ vector and pACT2-AIM23 plasmid (−/AIM23), (2) pAS2ΔΔ-PET111 plasmid and empty PACT2 vector (PET111/−), (3) pAS2ΔΔ-PET111 plasmid and pACT2-AIM23 plasmid (PET111/AIM23), (4) pAS2ΔΔ-AEP1 plasmid and pACT2-AIM23 plasmid (AEP1/AIM23). The names of the strains are depicted on the left. Left panel: growth on medium lacking histidine supplemented with the 3-amino-1,2,4-triazole (3-AT). Right panel: growth on the rich glucose-containing medium (YPD). The ten-fold consecutive dilutions of the yeast suspensions were plated. The experiment was done in three biological replicates; the characteristic picture is presented. (**C**) The results of the gel-shift experiment. In vitro synthesized *COX2* mRNA 5′-UTR was labeled with ^32^P-ATP and then incubated with different amounts of the recombinant Aim23p and/or Pet1119 (molar concentrations of the proteins are indicated on the top). Reaction products were separated using native PAGE and visualized by radioautography. The gel zones corresponding to low molecular weight (LMW) products, free UTR and UTR complex with Pet111p are marked by the arrows on the left. The asterisks mark two characteristic reactions where Pet111p concentration was equal, and one reaction contained Aim23p (complex detected) and another did not (complex not detected). The experiment was done in four biological replicates; the characteristic picture is presented.

We focused on the only known *COX2* mRNA translational activator, Pet111p, to understand how Aim23p could affect *COX2* mRNA translation in a UTR-dependent manner without binding them. This protein is a good candidate for being a mediator between the above-mentioned mRNA and Aim23p: it has been repeatedly demonstrated that Pet111p genetically interacts with the *COX2* mRNA [19,20], and recently physical interaction has been proven [25]. In this work, we tested physical interaction between Aim23p and Pet111p. For this, we used recombinant proteins separation by non-denaturing Polyacrylamide Gel Electrophoresis (PAGE) with imidazole-HEPES buffer system [26] (Figure 2A). We decided to use this buffer system for two reasons. First, the pH of gel here is 7.4 which is in good compliance with physiological conditions. Second, the calculated pI values of both proteins are higher than pH of the selected buffer system (10.6 for Aim23p and 9.06 for Pet111p, according to *Saccharomyces* genome database, https://www.yeastgenome.org), so these proteins should migrate to the cathode in the selected buffer system. Surprisingly, we did not detect free Pet111p (Figure 2A, lane 1). This could be explained by structural features of Pet111p or uneven charge distribution in this protein. However, the band of free Aim23p was clearly visible (Figure 2A, lane 2). Adding recombinant Pet111p to Aim23p led to the appearance of a shifted band on the native gel. Moreover, the exact mobility of this band depended on the amount of Pet111p added (Figure 2A, lanes 3–5). Thus, we can conclude that Aim23p physically interacts with Pet111p *in vitro*. The presence of the competitor (bovine serum albumin) ensured the specificity of this binding. 

The second method we used to investigate this interaction was the yeast two-hybrid system [27]. The principle of this assay is similar to that of the above-described three-hybrid system: one can detect the interaction between two proteins by demonstrating the growth of specially designed yeast strain on the medium without histidine. We tested the interaction between Aim23p and Pet111p, as well as between Aim23p and Aep1p, a protein that takes part in *ATP9* mRNA translation [28,29] (Figure 2B). Indeed, the strain containing *AIM23* and *PET111* genes grew faster than the strain containing *AIM23* only on the medium with 3-AT, which showed the interaction between these two proteins in the yeast cells. The strain containing Aim23p and Aep1p grew with the same rate as *AIM23*-only strain. The clear difference in the growth rates between *AIM23*/*PET111* and the other two strains tested suggests the absence of Aim23p interaction with Aep1p, which confirms the specificity of the assay. Finally, all of the strains grew on the glucose-containing full medium with the same rates (YPD on Figure 2B), which means that the observed difference in growth rates on the medium with 3-AT is not due to some general metabolic defects.

Thus, we demonstrated the physical interaction between Aim23p and Pet111p by two different methods, but the biological significance of this interaction remained unclear. We hypothesized that Aim23p might strengthen Pet111p interaction with 5′-UTR of *COX2* mRNA. To verify this, we again used the gel-shift approach. We purified recombinant Pet111p and first tested its interaction with 5′-UTR of *COX2* mRNA (Figure 2C, lanes 1–6). In line with the results by Jones et al. [25], we detected that the amount of UTR complex with Pet111p increased with the increase of the protein concentration. At the same time, the amount of the unbound UTR decreased, which pointed to the complex formation. Remarkably, adding recombinant Aim23p to the reaction led to the intensification of UTR•Pet111p complex formation (Figure 2C, lanes 7–10). The corresponding band was visible even if the Pet111p concentration was 0.05 µM; at this concentration, Pet111p alone was not able to form the complex with 5′-UTR of *COX2* mRNA (Figure 2C, lane 2). Thus, Aim23p seems to facilitate the Pet111p association with this UTR through direct protein-protein interaction.

### 2.4. The Increased Amount of Pet111p May Compensate for Aim23p Absence in the Process of COX2 mRNA Translation

**Figure 3:**
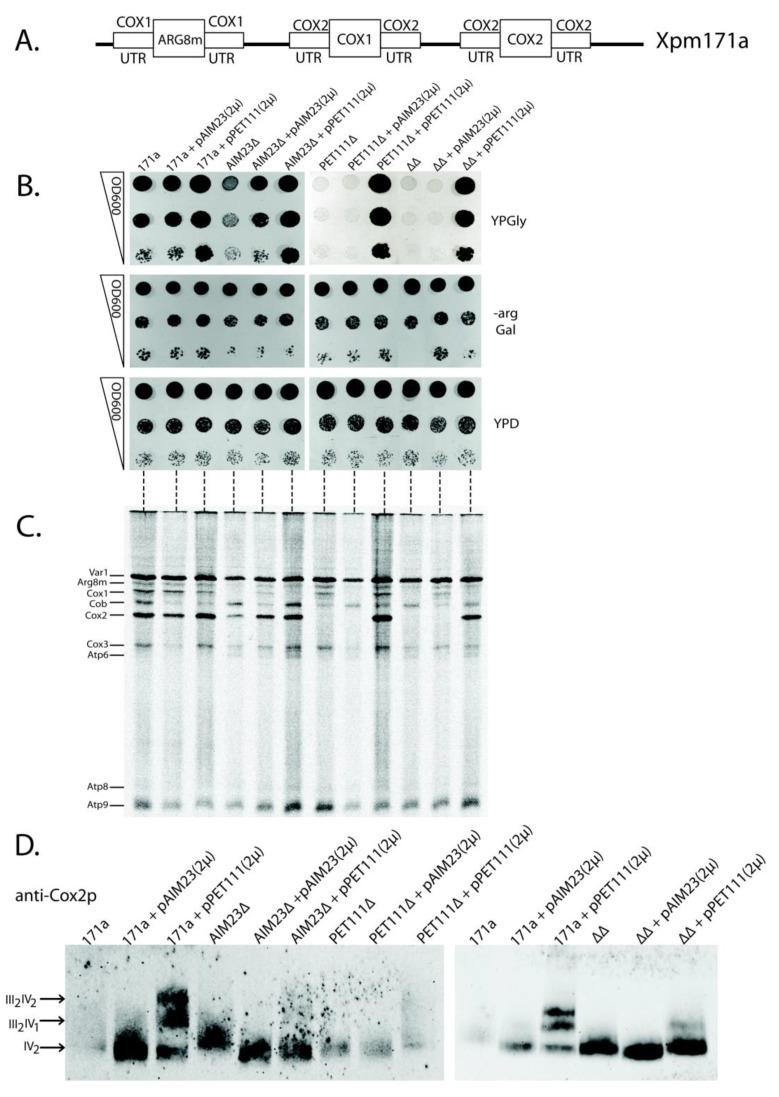
Increased Pet111p amounts ensure proper translation of *COX2* mRNA in the absence of Aim23p. (**A**) Scheme of the mitochondrial genome organization of XPM171a strain. (**B**) The pictures of 3-days yeast growth on the plates with different media (indicated on the right: YPD—full glucose-containing medium, -arg Gal—synthetic arginine-lacking medium with galactose, YPGly—full glycerol-containing medium). Strains are depicted on the top. 171a—XPM171a (parental strain for all other strains). AIM23∆—*AIM23* gene disrupted, PET111∆—*PET111* gene disrupted, ∆∆—both genes disrupted. pAIM23(2µ)—strain transformed with the two-micron plasmid bearing *AIM23* gene, PET111(2µ)—strain transformed with the two-micron plasmid bearing *PET111* gene. The ten-fold consecutive dilutions of the yeast suspensions were plated. The experiment was done in three biological replicates; the characteristic picture is presented. (**C**) Mitochondrial translation products in the yeast strains described in the legend to Figure 3B. Fractions of ^35^S-containing proteins (50 µg of total protein per lane) were PAGE-separated. Mitochondrially-encoded proteins were then visualized by radioautography. The order of mitochondrial proteins loading on the gel is the same as the order of plating the corresponding yeast strains on Figure 3B which is marked by horizontal dashed lines between two panels. Individual mitochondrial proteins are depicted on the left according to the original article where XPM171a strain has been created [31]. The experiment was done in three biological replicates; the characteristic picture is presented. (**D**) Analysis of the respiratory chain supercomplexes formation in the same yeast strains. Visualization was done by Western blot hybridization with antibodies against Cox2p after complexes separation by Blue Native PAGE. Designations of the strains are the same as in Figure 3B; they are indicated on the top. Supercomplexes with different stoichiometric ratios of complexes III and IV are indicated by arrows on the left. The experiment was done in three biological replicates; the characteristic picture is presented.

We then decided to test the possible functional links between Aim23p and Pet111p by the overexpression of the *PET111* gene in the presence or absence of Aim23p. This idea came from the fact that high expression of *PET111* led to an increase in the *COX2* mRNA translation [30]. Thus, a large amount of Pet111p in yeast mitochondria could potentially compensate the negative effects of Aim23p absence. We have chosen the yeast strain XPM171a to verify this hypothesis [31]. Figure 3A schematically represents the organization of mitochondrial DNA of this strain. Briefly, the UTRs of the COX1 mRNA control the expression of *ARG8m*, and the UTRs of the *COX2* mRNA control biosynthesis of both Cox1p and Cox2p. Such mitochondrial genome organization excludes the influence of *COX1* mRNA UTRs on mitochondrial function. On the contrary, any negative effect related to the *COX2* mRNA UTRs would lead to a drastic decrease of organelle functionality, since the biosynthesis of both Cox1p and Cox2p would weaken.

We made several genomic disruptions in XPM171a strain, which resulted in a series of four strains: parental XPM171a (“171a” on Figure 3B), XPM171a with *AIM23* disrupted (AIM23∆), XPM171a with *PET111* disrupted (PET111∆), and XPM171a with both genes disrupted (∆∆). Subsequently, we transformed each of these strains by the high copy number 2-micron plasmid YEplac195, either empty, or bearing *AIM23* gene (pAIM23(2µ) on Figure 3B), or *PET111* gene (pPET111(2µ) on Figure 3B). Finally, we checked the resulting strains for their growth rate on glycerol-containing medium as the sign of mitochondrial function. We represent these data as the “drop-tests” on the plates (Figure 3B), as well as the growth curves of the liquid cultures (Figure S1). The overproduction of Pet111p in parental XPM171a strain led to the increase of growth rate on glycerol medium (Figure 3B, upper panel, lanes 3, 6, 9, and 12), which was also seen earlier [30]. The deletion of the *AIM23* gene in the XPM171a strain resulted in growth delay, which showed abolished mitochondrial function (Figure 3B, upper panel, compare lanes 1 and 4). This was expected, since *AIM23* deletion in wild type yeast led to the same lag period in growth [13]. We detected the full restoration of this growth delay upon the overexpression of *AIM23* from the plasmid (Figure 3B, upper panel, lane 5). More interesting, the overexpression of *PET111* was able to compensate for the *AIM23* deletion in XPM171a strain (Figure 3B, upper panel, lane 9): the corresponding strain grew with the same rate as the parental strain. These data point to the fact that a higher amount of Pet111p is enough for the normal translation of *COX2* mRNA, even if there is no Aim23p in the mitochondria. This effect was even more pronounced in the double deletion strain, which was unable to grow on glycerol without rescuing plasmid (Figure 3B, upper panel, lanes 10–12). The overproduction of Pet111p from the plasmid compensated for this defect (Figure 3B, upper panel, lane 12). The normal, almost equally fast growth of all tested strains on the medium without arginine (Figure 3B, middle panel) ensures that neither Aim23p nor Pet111p affect the COX1 mRNA UTRs as *ARG8m* expression is under control of these UTRs (see Figure 3A). The only effect observed on this medium is the slight delay of the growth of the *AIM23*-lacking strains. This can be explained by the known Aim23p necessity for COX1 mRNA translation [13]. It is also clear that growth defects observed on YPGly medium were not due to some general metabolic disturbances since all the experimental strains grew with the same rate on glucose-containing medium (Figure 3B, lower panel). Overall, it is possible to state that the high amount of Pet111p makes yeast mitochondria competent in *COX2* mRNA translation without Aim23p. A possible explanation of this phenomenon is that Aim23p takes part in Pet111p recruitment to UTRs of *COX2* mRNA. The absence of Aim23p detains the normal interaction of Pet111p with these UTRs, which can be overcome by an excess of Pet111p in mitochondria.

We performed a direct analysis of mitochondrial protein synthesis in the above-mentioned strains to support this conclusion. For this, we inhibited their cytosolic translation by cycloheximide and then supplied the yeast cultures with ^35^S-methionine, which could only incorporate into the mitochondrially-synthesized proteins (methodological details are given in Materials and Methods). Figure 3C presents the results. The rates of Cox1p and Cox2p synthesis are of special interest, since both corresponding ORFs are under the control of *COX2* mRNA UTRs. Cox2p synthesis was slower in XPM171a strain with *AIM23* deletion when compared to the parental strain (Figure 3C, compare lanes 4 and 1), but the rate reached normal levels upon *PET111* overexpression (Figure 3C, lane 6). In double deletion strain, Cox2p was almost not synthesized (Figure 3C, lane 10); however, Pet111p overproduction led the complete restoration of Cox2p synthesis (Figure 3C, lane 12). This is in full accordance with the observed rates of growth on the glycerol-containing medium (Figure 3B). On the other hand, the situation with the Cox1p synthesis rate is more complex. We did not detect a direct correlation between this parameter and the rates of the growth of the corresponding strains on glycerol-containing medium. This might be explained by the fact that at least four translational activators participate in the regulation of COX1 mRNA translation, and at least one of them, Mss51p, interacts with the nascent polypeptide chain and the coding mRNA part [31]. Thus, we cannot exclude some additional effects on the Cox1p synthesis due to our genetic manipulations with the XPM171a strain, even if it is under control of *COX2* mRNA UTRs. Concerning Arg8m, its synthesis rate in the experimental strains generally depends on Aim23p presence or absence (Figure 3C), which has been expected, because Aim23p should control this protein synthesis in all XPM171a-derivative strains via *COX1* mRNA UTRs. Finally, Coomassie staining of the same gel showed that we loaded an equal amount of material on each lane (Figure S2). In summary, mitochondrial translation analysis supports our conclusion that Aim23p ensures Pet111p binding to the *COX2* mRNA UTRs, and that this binding is possible in the absence of Aim23p if Pet111p is overproduced.

Mitochondrial genome organization is non-canonical in the XMP171a strain, and this should lead to some quantitative imbalance in mitochondrial protein synthesis. This, in turn, might alter the process of the respiratory chain complexes assembly. The last stage of this process is the formation of supercomplexes—macromolecular structures consisting of several certain complexes in different stoichiometric ratios. The major supercomplexes in yeast mitochondria are those containing two complexes III and one complex IV (III_2_IV_1_) and two complexes III and two complexes IV (III_2_IV_2_) [32]. *PET111* overexpression leads to the increase of XMP171a strain growth rate on glycerol medium and the increase of the Cox2p synthesis rate, which could result in the intensified supercomplex assembly, as has been mentioned above. Thus, we decided to assess the supercomplex stoichiometry in our yeast strains. For that, we isolated mitochondria from all strains, solubilized them with digitonin, separated complexes by Blue Native polyacrylamide gel electrophoresis, and then visualized by staining with anti-Cox2p antibodies (Figure 3D). In the wild type XPM171a strain, we could only detect the dimers of complex IV. The defects of higher order structures assembly may result from the disturbance of the feedback loop mechanism of cytochrome oxidase assembly [16] due to abnormal organization of *COX1* and *COX2* mRNAs in the Xpm171a strain. The overexpression of *AIM23* did not alter supercomplexes composition, while Pet111p overproduction led to the formation of III_2_IV_1_ and III_2_IV_2_ supercomplexes (Figure 3D, compare lanes 3 and 1). We also observed a similar trend in the strains with *AIM23* and *PET111* genes disrupted (Figure 3D, lanes 6, 9, and 12). It is worth mentioning that, in the double deletion strain, the amount of complex IV dimes is notably higher than that in the parental Xpm171a strain. This might be attributed to a partial normalization of the feedback loop function upon the *AIM23* and *PET111* genes deletion, although the exact mechanism of such normalization is completely unclear. Coomassie staining of the same gel showed that we loaded an almost equal amount of material on each lane (Figure S2). Thus, *PET111* overexpression seems to normalize the processes of respiratory complexes assembly in XPM171a strain, which is in line with the observed increase of the rate of growth on glycerol-containing medium.

## 3. Discussion

This study was conducted to describe the mechanisms of mRNA-specific action of Aim23p in yeast mitochondrial translation. Being an ortholog of bacterial translation initiation factor 3, Aim23p was expected to be necessary for organellar protein biosynthesis. However, in 2016, we have shown that, among eight mitochondrially-encoded proteins, the synthesis of Cox1p and Cox2p goes less efficiently in the absence of this factor, Var1p, Cox3p, Cob1p, and Atp6p amount do not depend on Aim23p, and Atp8p and Atp9p biosynthesis rates increase in response to Aim23p absence [13]. Other research groups also detected similar misbalanced mitochondrial translation without Aim23p [33]. Moreover, in mice lacking MTIF3 (human mitochondrial initiation factor 3), misbalanced protein synthesis in mitochondria was also detected [34]. Finally, we have recently demonstrated that, in cultured HeLa cells, MTIF3 is dispensable for mitochondrial protein synthesis and the effect of its absence is not much pronounced on the level of individual mitochondrial proteins amount [35]. On the other hand, IF3 is absolutely required for translation in bacteria. It seems that mitochondrial IF3s, although being orthologous to bacterial IF3, have highly functionally diverged from the latter. One can hypothesize that, at least in yeast and in mammals, mtIF3 is primarily responsible for the maintenance of the proper relative rates of different mitochondrial proteins synthesis to ensure the correct assembly of the respiratory chain complexes.

In this work, we have chosen yeast *COX2* mRNA as one of the Aim23p targets. Using genetic experiments, we demonstrated that the Aim23p effect on this mRNA is mediated by the UTRs of the latter. However, we did not detect significant physical interaction between Aim23p and 5′-UTR of the *COX2* mRNA either in vitro (by gel-shift) or in vivo (by yeast three-hybrid system). This is in line with our initial idea that Aim23p might mainly play a regulatory role in mitochondrial translation. It is hard to imagine that this protein could exhibit so different effects on different mRNAs translation via direct binding to their UTRs. Instead, Aim23p might indirectly coordinate mitochondrial protein synthesis through some mRNA-specific translation factors. Yeasts are unique organisms where such factors (translational activators) exist; moreover, they mostly interact with UTRs of mitochondrial mRNAs. Thus, we tested the possible interaction of Aim23p with the only known activator of *COX2* mRNA translation, Pet111p, and showed that such interaction takes place. Moreover, Aim23p facilitates Pet111p binding to the 5′-UTR of *COX2* mRNA, as our gel-shift experiments have demonstrated. Aim23p might function as a necessary recruiter of Pet111p to its cognate mRNA, given that the amount of Pet111p (as well as all other translational activators) is as low as several hundred molecules per yeast cell [36]. In this case, one can assume that in a yeast strain with increased Pet111p amount, *COX2* mRNA translation could take place efficiently even without Aim23p. We demonstrate this exactly in the present work using specially designed yeast strain (XPM171a) where both Cox1p and Cox2p synthesis are under the control of *COX2* mRNA UTRs (see Figure 3A). In this strain, *COX1* mRNA UTRs had no influence on the mitochondrial function, which was very important to us: when manipulating with the *AIM23* gene, we were sure that any functional consequence would be mediated by *COX2* mRNA UTRs, but not by those of COX1 mRNA. Upon the deletion of *AIM23* in this strain, we observed some abnormalities in mitochondrial function; however, they were completely restored when *PET111* was overexpressed.

**Figure 4:**
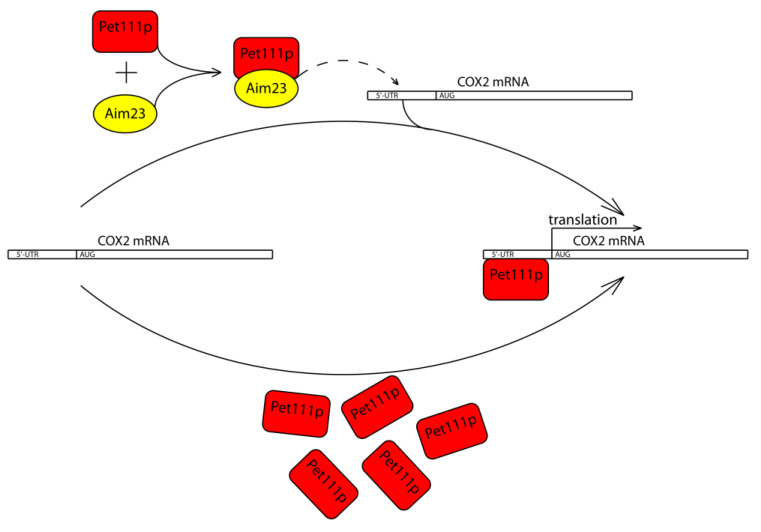
Working model of Aim23p involvement in the *COX2* mRNA translation. Aim23p does not seem to directly contact UTRs of this mRNA, or at least this potential contact plays secondary role in the whole process. Instead, Aim23p interacts with Pet111p. This interaction facilitates Pet111p binding to the 5′-UTR of the *COX2* mRNA, which, in turn, ensures its proper translation. In case when the amount of Pet111p is increased, no Aim23p is needed for activator interaction with *COX2* mRNA.

Taken together, all of the above-described results, we propose a working model of Aim23p involvement in the *COX2* mRNA translation (Figure 4). Aim23p does not seem to directly contact UTRs of this mRNA, or at least this potential contact plays secondary role in the whole process. Instead, Aim23p interacts with Pet111p. This interaction facilitates Pet111p binding to the 5′-UTR of the *COX2* mRNA, which, in turn, ensures its proper translation. In the case when the amount of Pet111p is increased, no Aim23p is necessary for activator interaction with *COX2* mRNA.

The amount of Cox1p also decreases in response to *AIM23* gene deletion [13]. Thus, one can propose that the mode of Aim23p involvement in Cox1p biosynthesis is similar to that of Cox2p. This means that Aim23p might physically interact with some of the *COX1* mRNA translational activators. This is rather possible given that many different activators (including those of *COX1* and *COX2* mRNAs) are simultaneously co-purified with yeast mitochondrial ribosomes [37]. It seems that translational activators might function together as a large macromolecular complex; the signs of such complex existence have been previously detected [38]. This hypothesis explains how Aim23p might affect the translation of *COX1* and *COX2* mRNAs at the same time. On the other hand, little experimental data exist so far in this field. A mechanism of Aim23p participation in *COX1* mRNA translation should undergo extensive studies. This will expand our vision of processes of translational activation and organization of its protein factors in yeast mitochondria.

## 4. Materials and Methods 

### 4.1. Plasmids, Strains, Oligonucleotides

Table 1, Table 2, and Table 3 depict the full lists of plasmids, strains, and oligonucleotides used, respectively.

No reference in the left columns of Table 1 and Table 2 means that the corresponding plasmid or strain has been constructed in this work.

### 4.2. Yeast Procedures

Yeasts were grown in one of the following media:

- YPD (20 g/L peptone, 10 g/L yeast extract, 20 g/L glucose),
- YPGly (20 g/L peptone, 10 g/L yeast extract, 30 g/L glycerol),
- SC-Arg (2.6 g/L amino acid mix without arginine, 3.4 g yeast nitrogen base, and 10 g/l ammonium sulfate) supplemented with 20 g/L galactose, and
- SC-His 3AT (2.6 g/L amino acid mix without histidine, 3.4 g yeast nitrogen base and 10 g/L ammonium sulfate) supplemented with 20 g/L glucose and 2 mM of 3-Amino-1,2,4-triazole (3-AT).

Yeast growth was analyzed by drop tests. For this, several ten-fold dilutions of the yeast suspensions were plated, starting from OD_600_ = 0.1. Yeast growth was also analyzed in the liquid medium. For this, the overnight yeast culture was diluted to the final OD_600_ = 0.1 in the appropriate medium and then incubated shaking at 30 °C in the wells of 24-well plate in Tecan Infinite Pro 200 (Tecan Group Ltd., Mannendorf, Switzerland) reader for 72 h. The values of OD_600_ in each well were calculated every 15 min. These values were plotted on the graph, together with the time points giving the growth curve.

### 4.3. Gene Deletions

The deletion of the *AIM23* gene was done via yeast native homologous recombination system. For this, the sequence of KanMX4 resistance cassette with 40 nt AIM23 5’- and 3’- flanks was amplified by PCR with the primers KanMX_aim23_mod_fw and KanMX_aim23_mod_rev while using the pAIM23-KanMX4 plasmid. The resulting product was introduced in yeast cells according to the standard transformation protocol using a mixture of polyethylene glycol and lithium acetate [46]. Transformants were selected on the plates containing YPD with 400 ug/mL G418. The presence of the intended deletion in the genomic DNA was confirmed by PCR with primers annealing upstream and downstream of the recombination junction sites. We used the pair of primers kanB/AIM23_A for the 5’-junction site and the pair of primers kanC/AIM23_D for the 3’-junction site.

*PET111* was deleted in an analogous way. The sequence of NatNT2 resistance cassette with 40 nt PET111 5’- and 3’- flanks was amplified by PCR with the primers ICO141 and ICO142 while using the plasmid pUG-natNT2 as a template. Transformants were selected on the plates containing YPD with 100 ug/mL nourseothricin. The presence of the intended deletion in the genomic DNA was confirmed by PCR with primers annealing upstream and downstream of the recombination junction sites. We used the pair of primers NAT_B/PET111_A for the 5’-junction site and the pair of primers NAT_C/PET111_D for the 3’-junction site.

### 4.4. Construction of YEplac195-Derivative Plasmids

The DNA fragment containing the *AIM23* gene that was flanked by 482 bp 5’-upstream and 246 bp 3’-downstream regions was amplified from yeast genomic DNA by PCR with the primers Aim23_YE_Fw and AIM23_YE_Rv. This fragment was inserted in the YEplac195 vector between HindIII and SalI sites.

The DNA fragment containing *PET111* gene flanked with 477 bp 5’- upstream and 238 bp 3’- downstream regions was amplified from yeast genomic DNA by PCR with the primers Pet111_YE_Fw and Pet111_YE_Rv. This fragment was inserted in YEplac195 vector between KpnI and BamHI sites.

### 4.5. Yeast Two- and Three-Hybrid Systems

The coding sequence of the *AIM23* gene was amplified by PCR with the primers AIM23_PACT2_Fw and AIM23_PACT2_Rv and then inserted in pACT2 vector between NcoI and XhoI sites in frame with the activation domain of GAL4. The expression of this vector (pACT2-AIM23) in yeast produced the Aim23p fused with the activation domain of the GAL4 transcription factor. 

The sequence of *PET111* gene was amplified by PCR with the primers PET111_pAS2_Fw and PET111_pAS2_Rv and then inserted in pAS2ΔΔ vector between BamHI and PstI sites in frame with the DNA binding domain of GAL4 at the N-terminus (pAS2ΔΔ-PET111).

The sequence of *AEP1* gene was amplified by PCR with the primers AEP1_pAS2_Fw and AEP1_pAS2_Rv and then inserted in pAS2ΔΔ vector [42] between BamHI and PstI sites in frame with the DNA binding domain of GAL4 at the N-terminus (pAS2ΔΔ-AEP1).

The DNA sequence of the 5′-UTR of the *COX2* mRNA [47] was amplified by PCR from yeast total DNA with the primers pIIIA_UTR_Fw and pIIIA_UTR_Rv and then inserted into the at SmaI restriction site. The resulting construct (pIIIA-UTR) produced the 5′-UTR of the *COX2* mRNA fused with two stem-loops binding the MS2 phage coat protein.

The constructs pACT2-AIM23, pAS2ΔΔ-PET111, and pAS2ΔΔ-AEP1 in different combinations were transformed in the Y190 yeast strain. The constructs pACT2-AIM23 and pIIIA-UTR in different combinations were transformed in the L40 coat yeast strain. For the transformation, PEG-LiAc protocol was used [46]. The positive Y190 transformants were selected on the medium without tryptophan and leucine; the positive L40 coat transformants were selected on the medium without leucine and uracil. The transcription of the *HIS3* gene is activated in the case of the successful interactions between two fusion proteins or between fusion protein and hybrid RNA. We assayed the expression of the *HIS3* gene in the transformants by plating them on the SC-His medium containing 2 mM of 3-Amino-1,2,4-triazole, a competitive inhibitor of the *HIS3* gene product. For this, the consecutive ten-fold dilutions of the yeast cultures of the Y190 strain transformed with different plasmids starting from OD_600_ of 0.1 were plated.

### 4.6. Expression and Purification of Recombinant Proteins

Recombinant Pet111p was expressed and purified, as described in [25], with minor modifications. The sequence of *PET111* without mitochondrial targeting sequence was amplified by PCR with the primers ICO61 and ICO62 and inserted in pET21d vector between NheI and XhoI sites. The construct was transformed in the B834 *E. coli* strain. The bacteria were grown in 500 mL of LB supplemented with 50 μg/mL ampicillin up to OD_600_ = 0.8. Subsequently, the production of the protein was induced by the addition of 10 μM IPTG with subsequent incubation at 12 °C overnight. Bacteria were collected by centrifugation at 3000g for 10 min, washed with PBS, resuspended in 10 mL of buffer A (20 mM Tris-HCl pH 7.5, 500 mM NaCl, 30 mM imidazole) supplemented with EDTA-free protease inhibitor cocktail, and sonicated six times for 10 s with 30 s intervals at 25% amplitude (using Branson digital sonifier, Branson Ultrasonics, Brookfield, CT, USA). The lysate was clarified by centrifugation at 30,000× *g* for 30 min and applied at HisTrap 1 mL column connected to the AKTA Purifier FPLC system (GE Healthcare, Chicago, IL, USA). The column was washed with 25 volumes of buffer A, and recombinant Pet111p was eluted with buffer B (20 mM Tris-HCl pH 7.5, 500 mM NaCl, 300 mM imidazole). Pet111p was transferred to storage buffer (PBS supplemented with 20% glycerol) while using desalting column and stored at −20 ^°^C

The expression and purification of Aim23p were done according to the previously described procedure [6].

### 4.7. Gel-Shift Assay

*COX2* mRNA 5′-UTR was obtained in in vitro transcription reaction by T7 RNA polymerase (Thermo Fisher Scientific, Waltham, MA, USA) with annealed corresponding oligonucleotides as the template. After the reaction, the mix was digested with DNAse I (Thermo Fisher Scientific, USA) and then loaded on 8% denaturing PAAG. After electrophoresis, band corresponded to *COX2* mRNA 5′-UTR was excised, and RNA was passively eluted in 1 mL 20 mM Tris-HCl pH 7.5, 250 mM NaOAc, 1 mM EDTA, and 0.25% SDS overnight at room temperature. Subsequently, RNA was precipitated, dephosphorylated by FastAP phosphatase (Thermo Fisher Scientific, USA), and then labeled with γ-^32^P-ATP with polynucleotide kinase (Thermo Fisher Scientific, USA).

Binding reactions were performed in 20 µL of 20 mM Tris-HCl pH 7.2, 50 mM NaCl, 5 mM MgCl_2_, 5% glycerol, each reaction mix contained 50 fmol of labeled *COX2* mRNA 5′-UTR, 0.5 pmol of unlabeled total yeast tRNA, and different concentrations of recombinant Pet111p and/or Aim23p. After binding for 20 min at room temperature, the reaction mixes were loaded on 8% PAAG at 1× TBE and separated at 100 V for 1 h. Afterwards, the gels were dried and subjected to autoradiographic analysis using Storm 865 scanner (GE Healthcare, USA).

### 4.8. Mitochondrial Translation Analysis

The labeling of yeast mitochondrially-synthesized proteins with ^35^S-methionine was carried out in whole cells that were cultured in medium containing 2% galactose as carbon source up to 2–3 units of OD_600_. The cells were incubated for 5 min at 30 °C in the presence of 0.2 mg/mL cycloheximide in order to inhibit cytosolic translation. Immediately after this, 25–30 µCi of ^35^S-methionine (Perkin Elmer, Waltham, MA, USA) was added, and incubation continued for 20 more minutes at 30 °C. After incorporation of the label into the products of mitochondrial translation, 50 µg of total cell proteins per lane were separated on a 17.5% PAAG and then subjected to autoradiographic analysis while using Storm 865 scanner (GE Healthcare, USA).

### 4.9. Mitochondria Isolation

Mitochondria were isolated, as described in [48], with minor modifications. Briefly, the yeasts were grown in YPGal medium to OD_600_ ~2, collected by centrifugation at 4000 × *g* for 10 min, washed twice with water, and then resuspended in DTT buffer (0.1 M Tris-H_2_SO_4_, 10 mM DTT) at 2 mL/g wet weight ratio. After 30 min incubation at 30 °C, the cells were collected by centrifugation, washed with zymolyase buffer (20 mM K-phosphate buffer pH 7.4 and 1.2 M sorbitol) at 7 mL/g wet weight ratio, and then resuspended in the same volume of zymolyase buffer with 1 mg/g Zymolyase 20T. After incubation for 1 h at 30 °C and 80 rpm in the orbital shaker, spheroplasts were collected by centrifugation and washed again with zymolyase buffer at the same ratio. Subsequently, spheroplasts were resuspended in homogenization buffer (10 mM Tris-HCl pH 7.4, 0.6 M sorbitol, 1 mM EDTA, 1 mM PMSF, and 0.2% BSA) and then disrupted in Dounce homogenizer (15 strokes). Lysates were centrifuged at 3000× *g* for 10 min, and the crude mitochondrial fraction was sedimented at 17,000× *g* for 15 min. The pellet was gently resuspended in the minimal volume of SEM buffer (10 mM MOPS-KOH pH 7.2, 250 mM sucrose, and 1 mM EDTA), and was then applied at 15%/23%/32%/60% sucrose step gradient for ultracentrifugation at 134,000× *g* for 1 h. The pure mitochondrial fraction from interphase between 60% and 32% of sucrose was collected, diluted thrice with SEM buffer, and then sedimented at 17,000× *g* for 15 min. The mitochondria samples were stored at −80 °C.

### 4.10. Blue Native Polyacrylamide Gel Electrophoresis (PAGE)

Blue Native PAGE was performed, as described in [49], with minor modifications. About 100 µg of mitochondria were solubilized in 3% digitonin at 1:6 (total mitochondrial protein: digitonin) ratio for 30 min on ice, centrifuged at 30,000× *g* for 30 min, and then applied to 4–10% Blue native PAGE. After electrophoresis, the separated protein complexes were transferred to the Protran nitrocellulose membrane (GE Healthcare, USA) and stained with rabbit antibodies to Cox2p (kind gift from Ott M.) and Amersham ECL Rabbit IgG, HRP-linked whole Ab (NA934, GE Healthcare) secondary antibodies. The membranes were then stained with SuperSignal West Pico PLUS Chemiluminescent Substrate (Thermo Fisher Scientific, USA) and the signals were scanned with ChemiDoc instrument (BioRad, Hercules, CA, USA).

## Figures and Tables

**Table 1:** Plasmids used in the study.

Name, Reference	Description
pAIM23-KanMX4 [13]	pRS416 plasmid containing KanMX4 cassette with *AIM23* 5’- and 3’- flanks. Constructed in our lab previously.
pUG-natNT2 [39]	Plasmid containing NatN2 cassette. A gift from D.Knorre.
YEplac195 [40]	Shuttle episomal vector with 2-micron replication origin. A gift from D.Knorre.
pAIM23(2µ)	YEplac195 vector with *AIM23* gene and its genomic flanks cloned.
pPET111(2µ)	YEplac195 vector with *PET111* gene and its genomic flanks cloned.
pACT2 [41]	Vector for 2- and 3-hybrid systems. A gift from N.Entelis.
pACT2-AIM23	pACT2 with *AIM23* gene cloned.
pAS2ΔΔ [42]	Vector for 2-hybrid system. A gift from N.Entelis.
pAS2ΔΔ-PET111	pAS2ΔΔ with *PET111* gene cloned.
pAS2ΔΔ-AEP1	pAS2ΔΔ with *AEP1* gene cloned.
pIIIA/MS2-1 [43]	Vector for 3-hybrid system. A gift from N.Entelis.
pIIIA-UTR	pIIIA/MS2 with 5′-UTR of the *COX2* mRNA cloned.
pET21d [44]	Vector for recombinant protein expression in *E. coli.* A gift from V.Hauryliuk.
pET21d-PET111	pET21d with *PET111* gene cloned.
pET28a-AIM23 [6]	pET28a vector with *AIM23* gene cloned. Constructed in our lab previously.

**Table 2:** Strains used in this study.

Strain	Genotype/Description
*Saccharomyces cerevisiae*	
XPM171a [31]	Matα*, lys2, leu2-3,112, arg8::hisG, ura3-52* [*ρ^+^, cox1∆::ARG8m, cox2D::COX1^c^, COX2*]. A gift from M. Ott.
∆AIM23	XPM171a, *AIM23* gene disrupted with KanMX4 cassette.
∆PET111	XPM171a, *PET111* gene disrupted with NatNT2 cassette.
∆∆	XPM171a with *AIM23* and *PET111* genes disrupted with the above-mentioned cassettes.
Y190 [41]	*MATa gal4 gal80 his3 trp1–901 ade2–101 ura3–52 leu2–3, 112 URA3*∷*GAL1*∷*lacZ LYS2*∷*GAL4(UAS)*∷*HIS3 cyh*^R^ Vector for 2-hybrid system. A gift from N. Entelis.
L40 coat [24]	*MATa, ura3-52, leu2-3, 112, his3-200, trp1-1, ade2, LYS2::(lexA op)-HIS3, URA3::(lexA op)-lacZ, LexA MS2 coat (TRP1)* Vector for 3-hybrid system. A gift from N. Entelis.
*Escherichia coli*	
B834 [45]	*hsdS metE gal ompT* For recombinant proteins expression. A gift from D.Knorre.

**Table 3:** Oligonucleotides used in the work. All synthesized by Evrogen (Russia).

Name	Sequence
AEP1_pAS2_Fw	5′-ATGCGGATCCATGATTACTACAGTG
AEP1_pAS2_Rv	5′-ATGCCTGCAGTTATGGGCGTAAAGCTTC
AIM23_A	5’-TGGGTGTTGATA
AIM23_D	5’-TAGTATGGATGA
AIM23_PACT2_Fw	5′- ATGCCCATGGATGTTAAAAGTTCC
AIM23_PACT2_Rv	5′-ATGCCTCGAGTTACATTTCATTCATTT
Aim23_YE_Fw	5′-ATGCAAGCTTCCTCGTGTAAATGAAATCAAAGAGG
AIM23_YE_Rv	5′-ATGCGTCGACCATGCTCATAAATCCTGAC
ICO141	5’-CACTACCTATTAAATTTAAACAATTGCTTACGAGAACTTAGACATGGAGGCCCAGAATACC
ICO142	5’-ATTTACACGTGAGAGAAAGGAAGGTAAATAACTGAAAAGACCGGTAGAGGTGTGGTCAATAAGAGC
ICO61	5′-TTGCGCTAGCACTGAGTTGATCAAAAAAAAGC
ICO62	5′-AAGCCTCGAGCTCCTCCTCCTTTTTATTCTC
kanB	5’-CTGCAGCGAGGAGCCGTAAT
kanC	5’-TGATTTTGATGACGAGCGTAAT
KanMX_aim23_mod_fw	5’-CCCGCGACGGTAAGAACTTTA
KanMX_aim23_mod_rev	5’-GAATCCTGGTACTTTAATGATAAG
NAT_B	5’-ATGCCCCTGAGCTGCGCACG
NAT_C	5’-GAGTAACTCTTTCCTGTAGG
PET111_A	5’-GTACATTTGTTGAAGGAG
PET111_D	5’-GCCAATCAAGTACTGCC
PET111_pAS2_Fw	5′-ATGCGGATCCATGTTACAACGGAG
PET111_pAS2_Rv	5′-ATGCCTGCAGTTACTCCTCCTCCTTTTT
Pet111_YE_Fw	5′-ATGCGGATCCCCGGACCTTACGAGTTCTTCG
Pet111_YE_Rv	5′-ATGCGGTACCGCCCTCCTTCAAACTATTCG
pIIIA_UTR_Fw	5′-ATGCGGGCCCAGTATTAACATATTATAAATAG
pIIIA_UTR_Rv	5′-ATGCGGGCCCTTTAATAAATCTTAACC

## Supplementary Materials

Supplementary materials can be found at https://www.mdpi.com/1422-0067/21/10/3414/s1.
