# Supplementary material for: Yeast Mitochondrial Translation Initiation Factor 3 Interacts with Pet111p to Promote COX2 mRNA Translation
Source: Int J Mol Sci. 2020 May 12;21(10):3414. doi: 10.3390/ijms21103414 (PMC7279496; doi:10.3390/ijms21103414)
Supplement: Supplementary file 1 [file ijms-21-03414-s001.pdf]

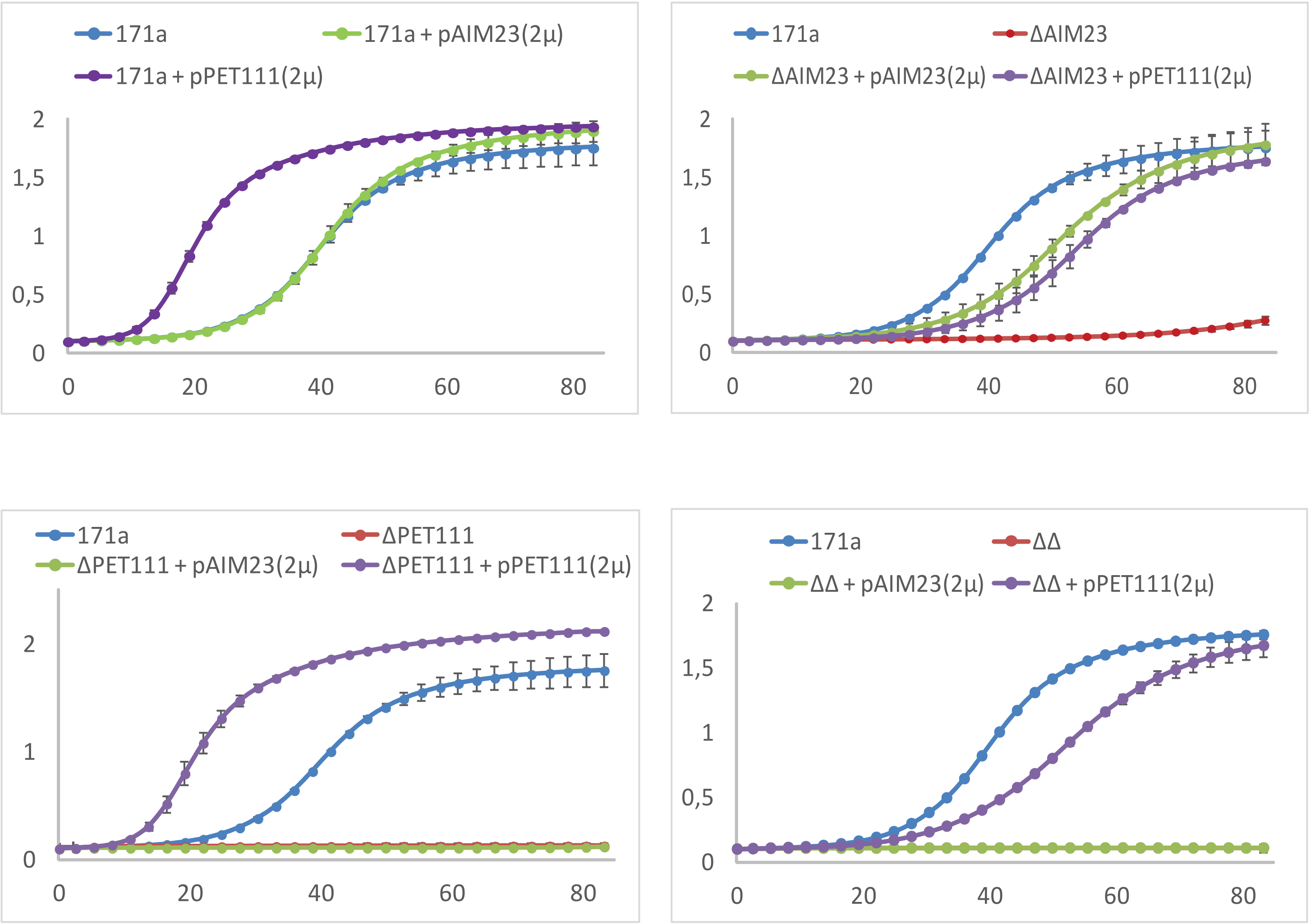

Figure S1. Curves of growth of the yeast strains described in the legend to Figure 3B in the glycerol-containing liquid medium. The color code is deciphered above each graph. Designations of the strains are the same as in Figure 3B. On X-axes: growth time, hours. On Y-axes: OD600, absorbance units. The experiment was done in three biological replicates; the characteristic picture is presented.

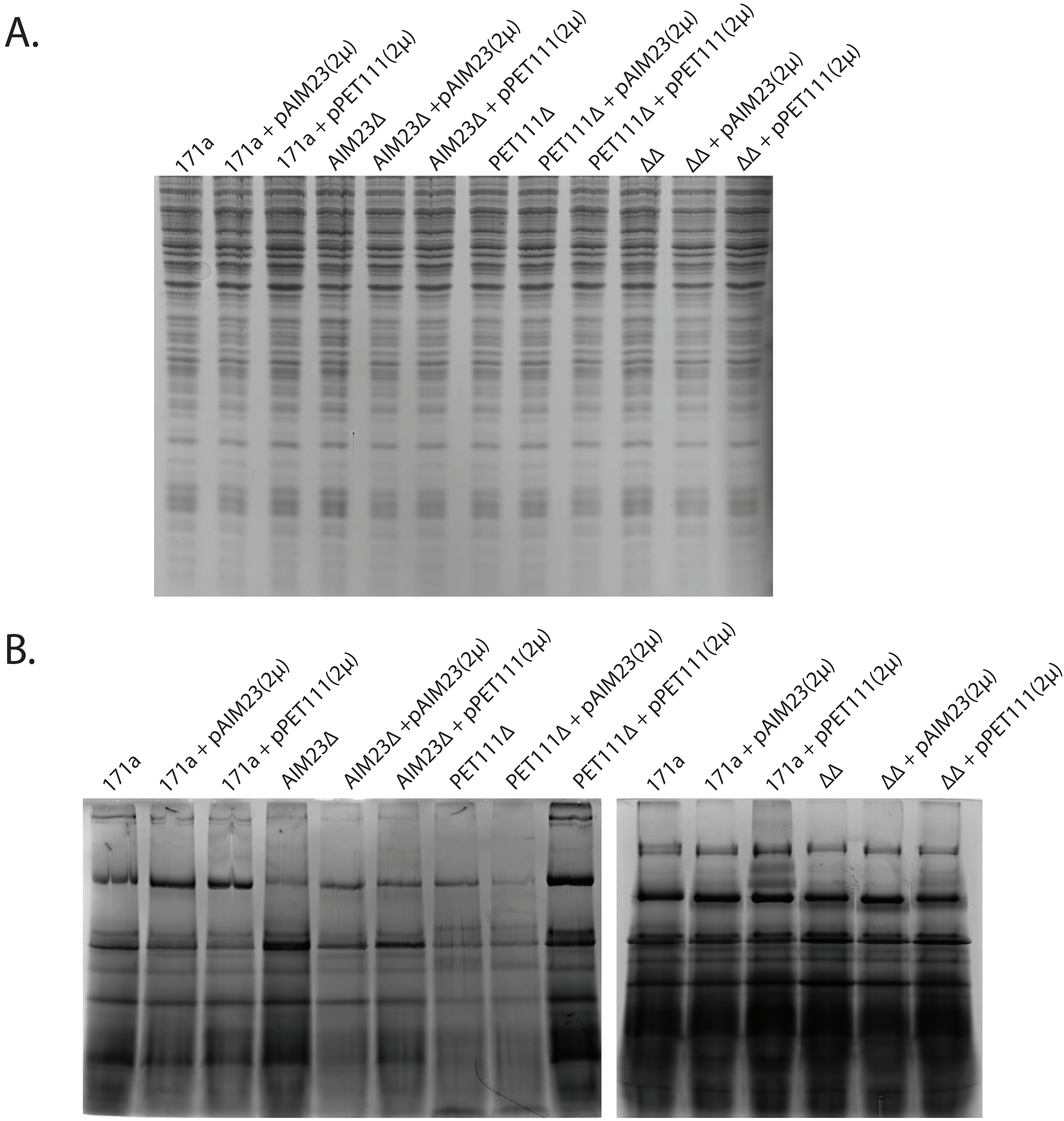

Figure S2. (a) Picture of the gel from Figure 3C stained with Coumassie. (b) Pictures of the gels from Figure 3D stained with Coumassie.

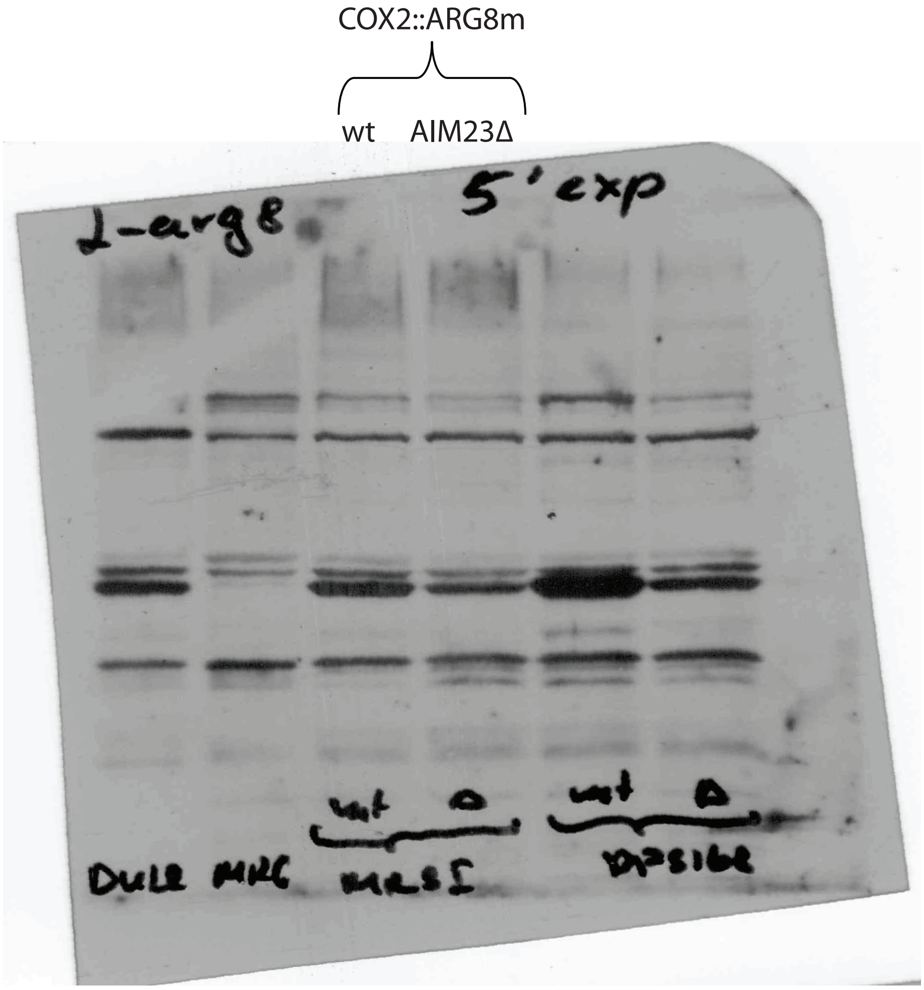

Figure S3. Original, uncropped and unadjusted image of the blot in Figure 1C (upper panel, Western blot hybridization with antibodies against Arg8p).

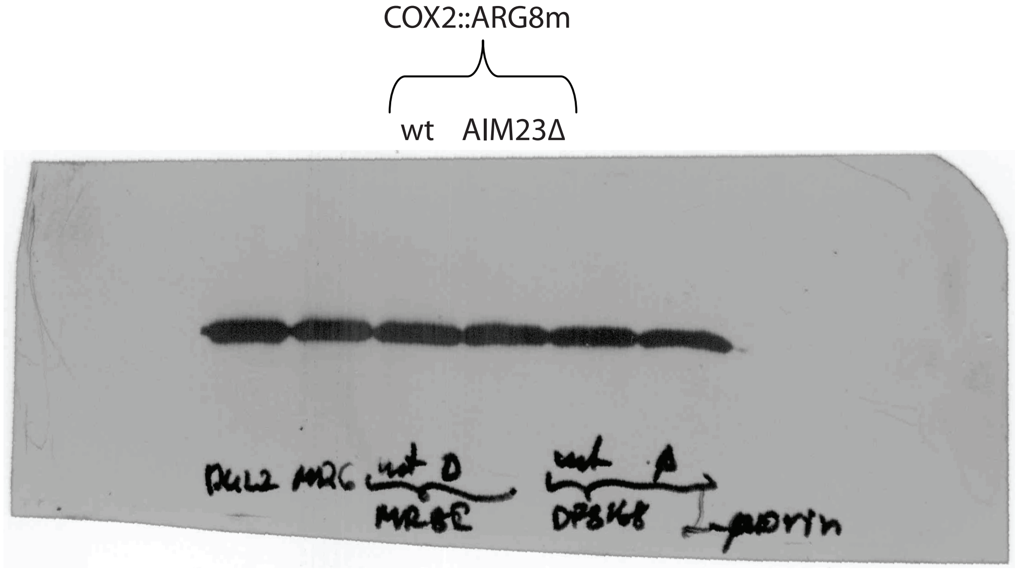

Figure S4. Original, uncropped and unadjusted image of the blot in Figure 1C (lower panel, Western blot hybridization with antibodies against Por1p).

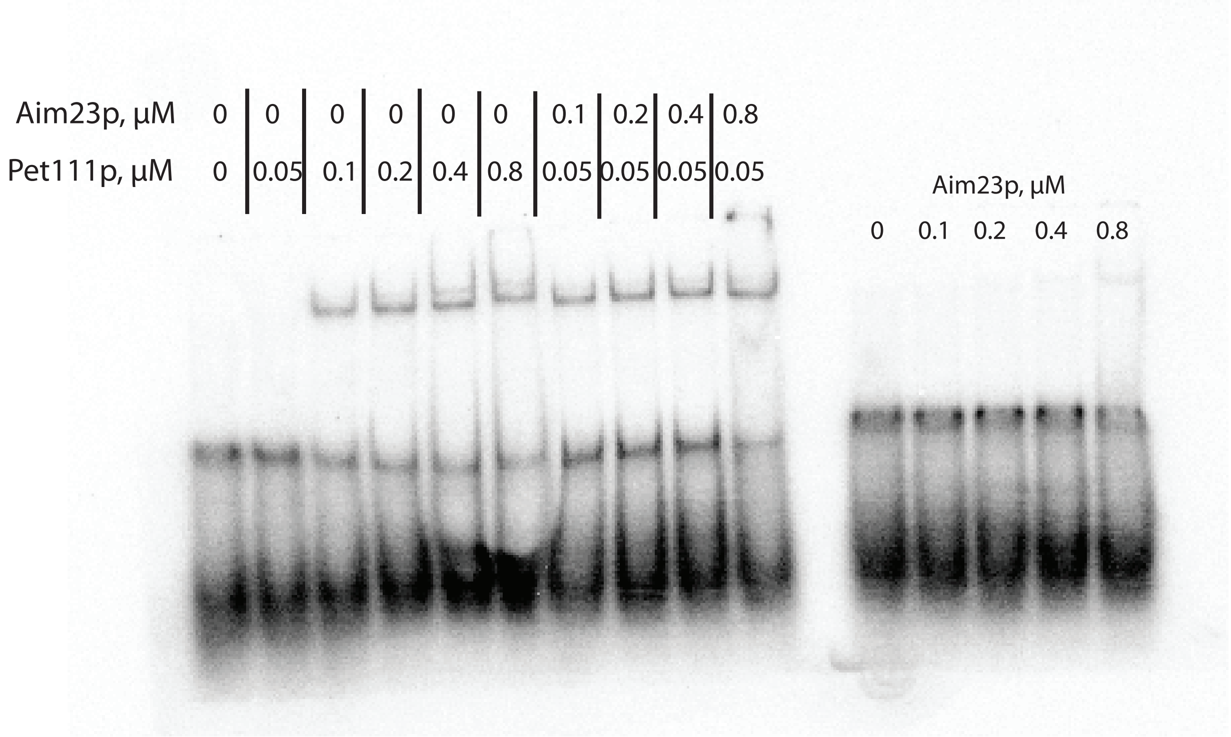

Figure S5. Original, uncropped and unadjusted image of the gel in Figure 1D (five right lanes of the gel) as well as in Figure 2C (ten left lanes of the gel).

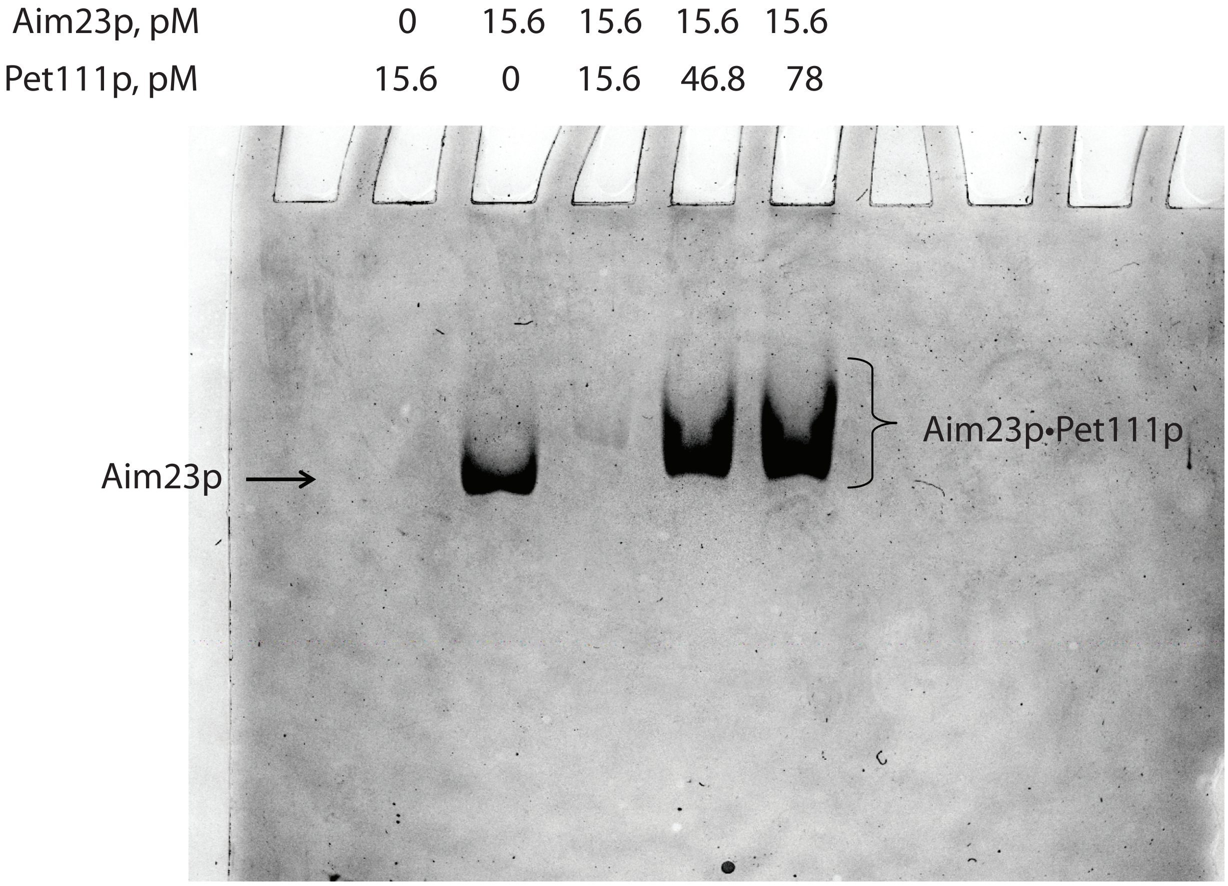

Figure S6. Original, uncropped and unadjusted image of the gel in Figure 2A.

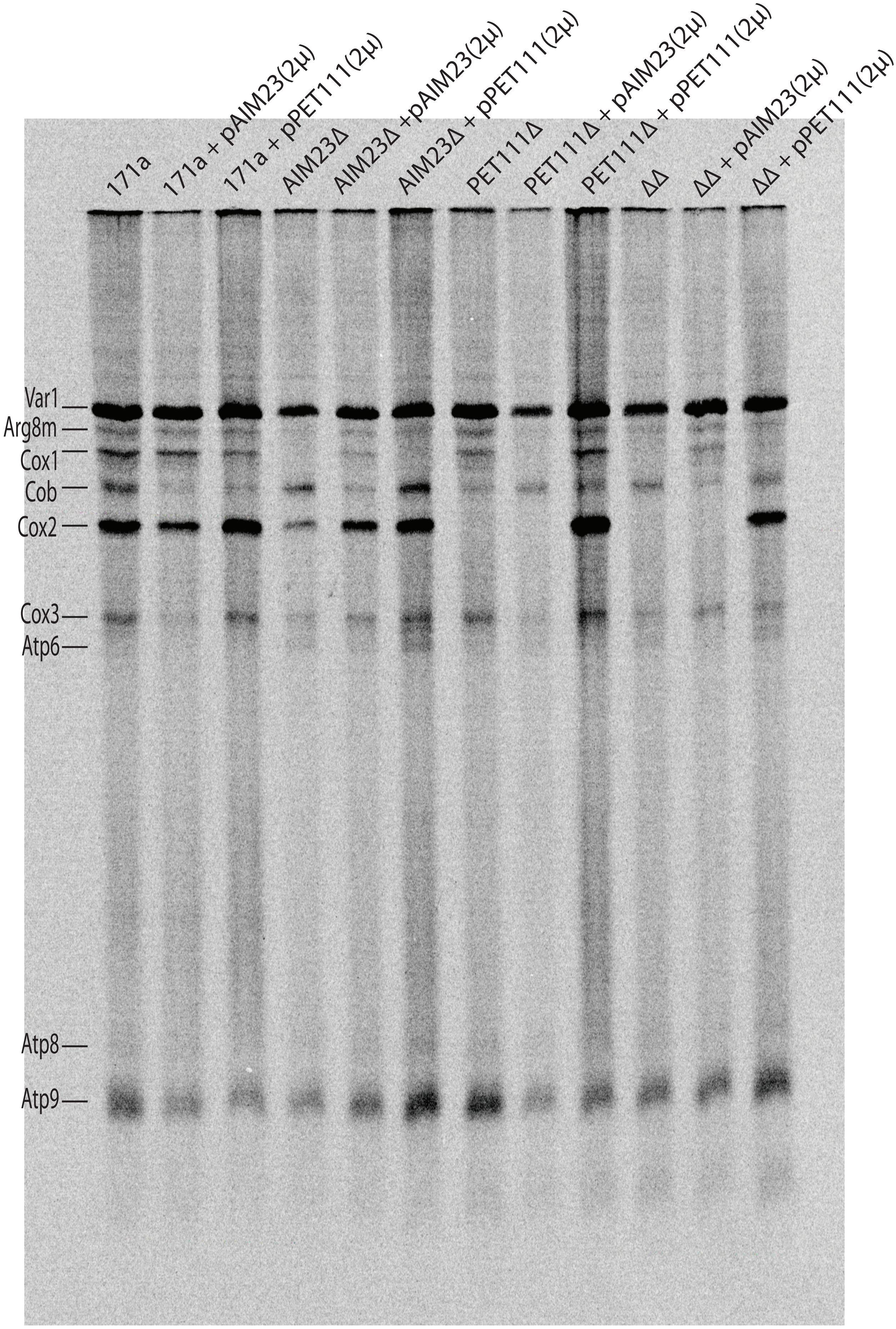

Figure S7. Original, uncropped and unadjusted image of the gel in Figure 3C.

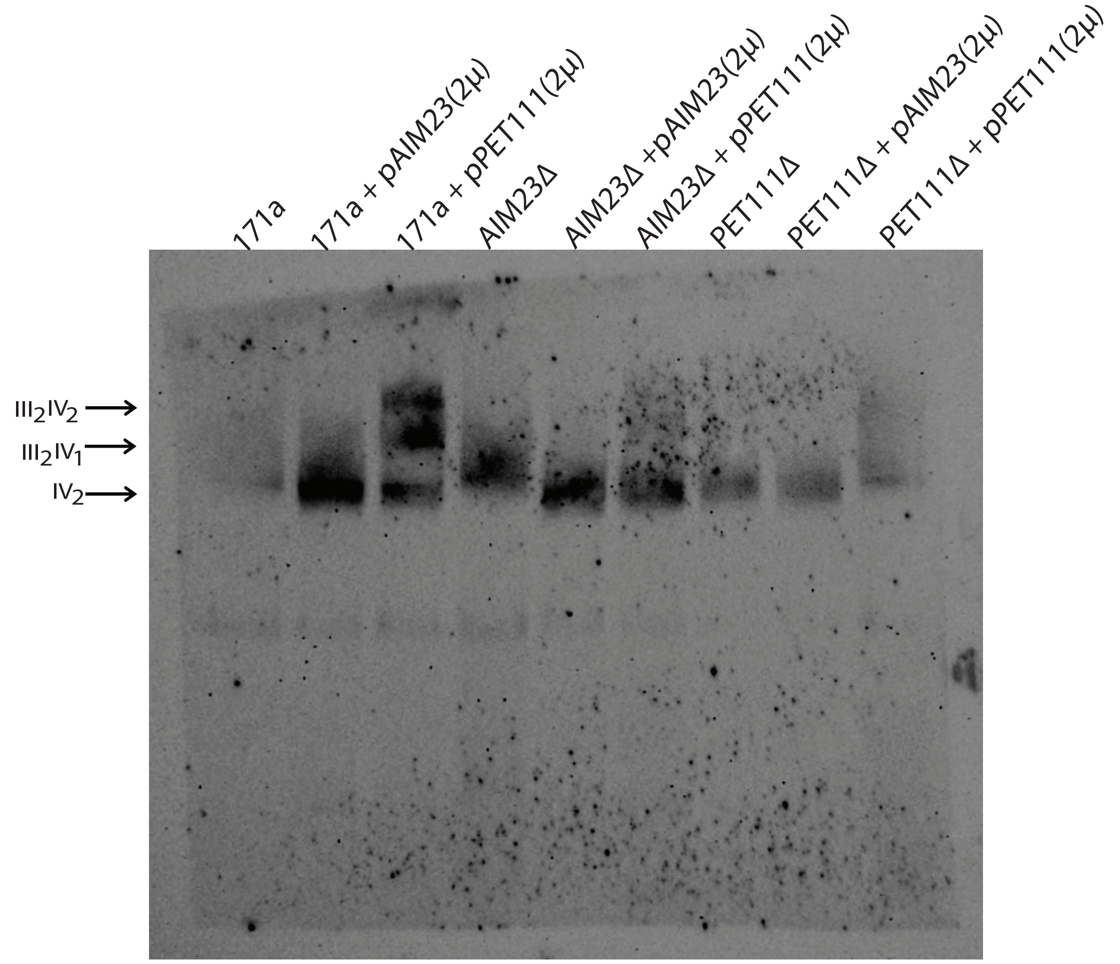

Figure S8. Original, uncropped and unadjusted image of the blot in Figure 4B (left panel).

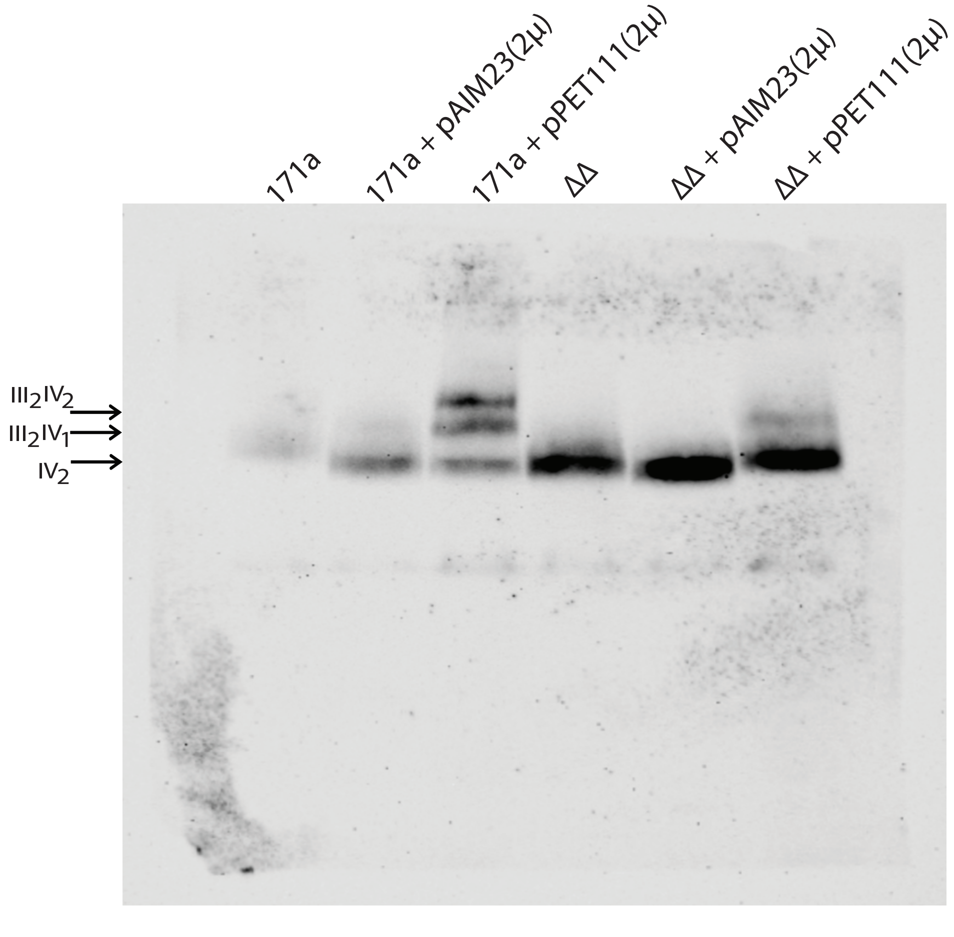

Figure S9. Original, uncropped and unadjusted image of the blot in Figure 4B (right panel).

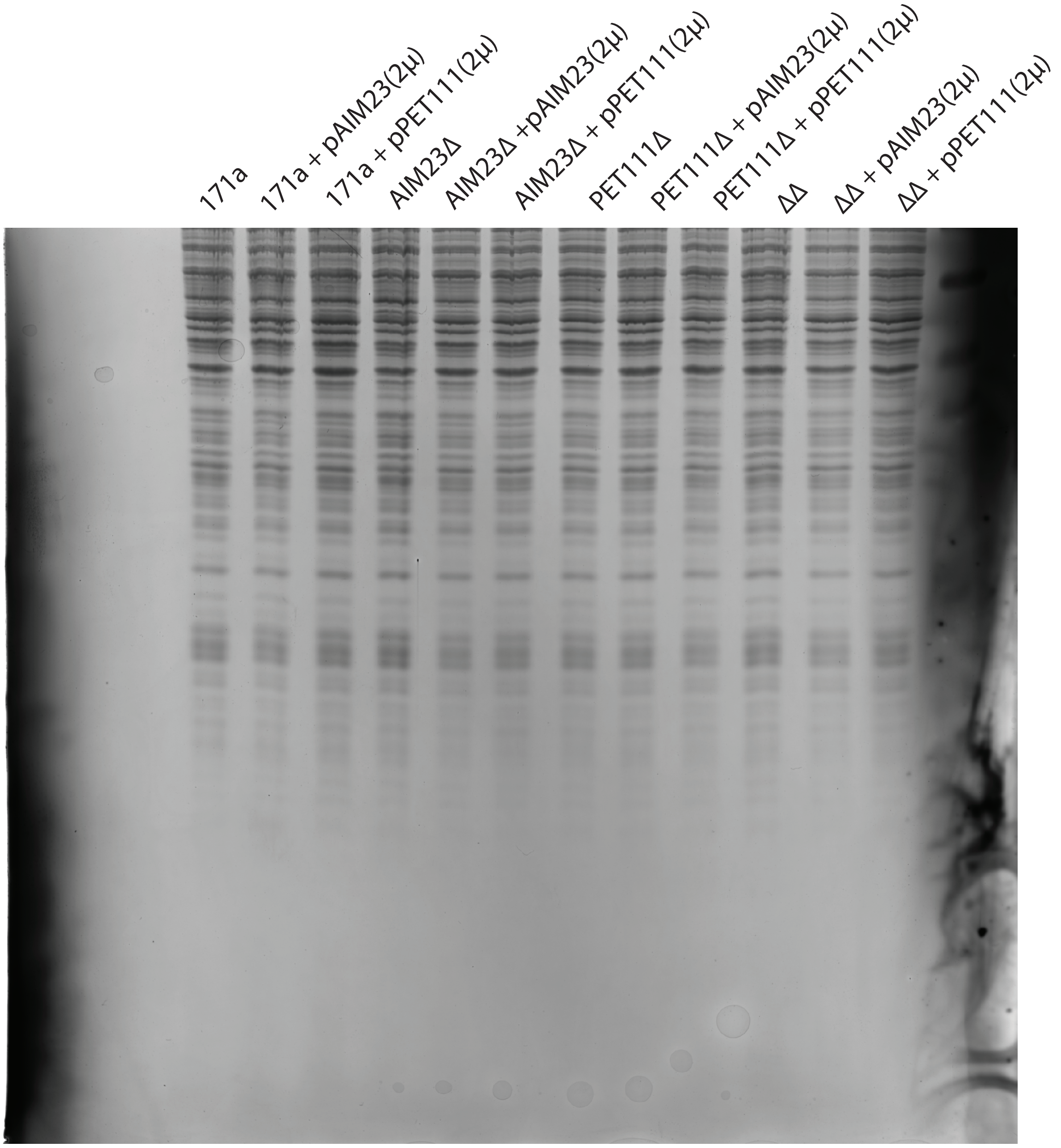

Figure S10. Original, uncropped and unadjusted image of the blot in Figure S2A.

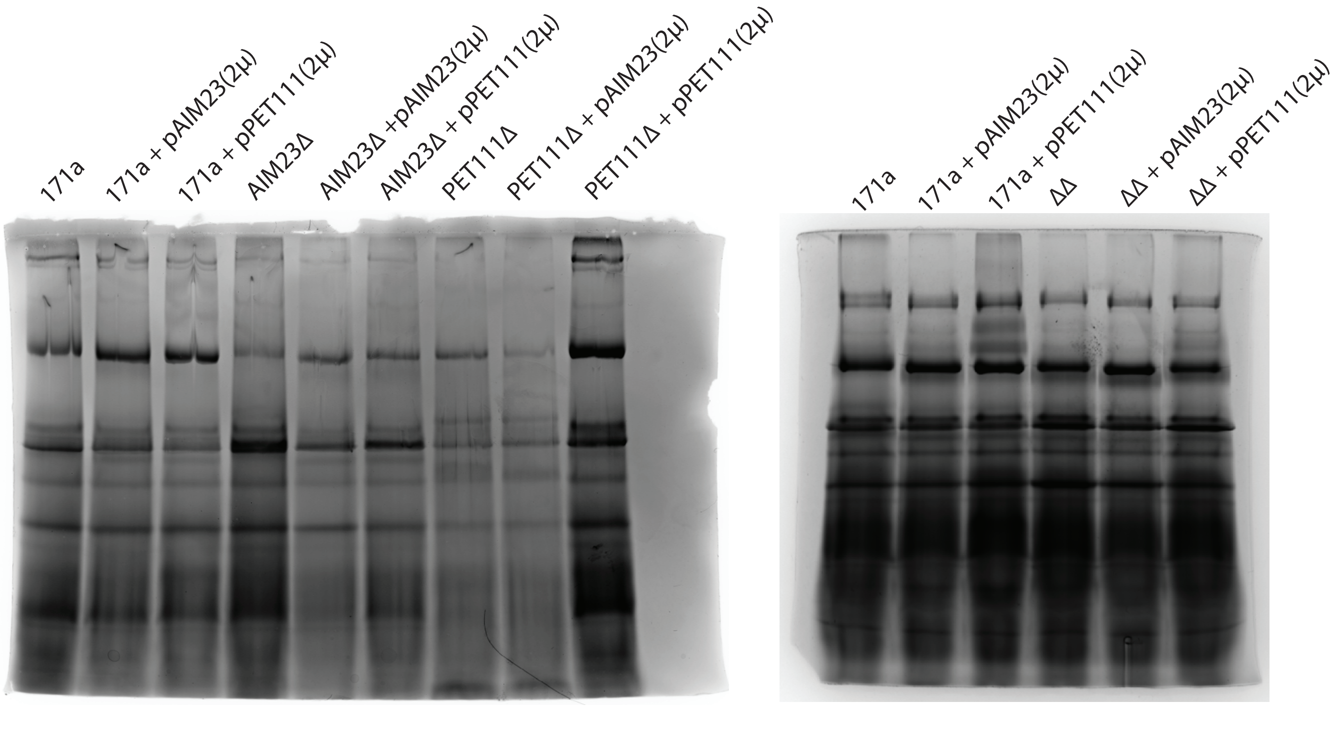

Figure S11. Original, uncropped and unadjusted images of the blot in Figure S2B.
